# Moral Injury in Gaza: The Impact on Healthcare Professionals

**DOI:** 10.1002/hsr2.72252

**Published:** 2026-04-16

**Authors:** Bilal Irfan, Abdulwhhab Abu Alamrain, Khalil Alinaby, Hasan Hussein, Abdallah Abu Shammala, Mohammed Halimy, Majdi Al‐Khaldi, Alaa Jamal Kassab, Fahmy Almedana, Hitham Ibrahim Toman, Basel Tarab, Roberto Sirvent, Abd Al‐Karim Sammour, Hanaa Mousa, Belal Aldabbour, Izzeddin Lulu, Ali Ghaleez, Amna Abusamhadana, Abdullah Ghali, Muna Irfan

**Affiliations:** ^1^ Center for Bioethics, Harvard Medical School Boston Massachusetts USA; ^2^ Center for Surgery and Public Health, Brigham & Women's Hospital Boston Massachusetts USA; ^3^ Department of Neurology University of Michigan Medical School Ann Arbor Michigan USA; ^4^ Department of Epidemiology University of Michigan School of Public Health Ann Arbor Michigan USA; ^5^ Al‐Aqsa Martyrs' Hospital Deir Al‐Balah Palestine; ^6^ Faculty of Medicine Al‐Quds University Jerusalem Palestine; ^7^ Faculty of Medicine Al‐Azhar University Gaza Palestine; ^8^ Harvard University Cambridge Massachusetts USA; ^9^ European Gaza Hospital Khan Yunis Palestine; ^10^ Faculty of Medicine Islamic University of Gaza Gaza Palestine; ^11^ Ain Shams University Cairo Egypt; ^12^ Winchester Hospital Winchester Massachusetts USA; ^13^ Boston College Chestnut Hill Massachusetts USA; ^14^ Department of Global Health and Social Medicine Harvard Medical School Boston Massachusetts USA; ^15^ Nasser Medical Complex Khan Yunis Palestine; ^16^ Al‐Shifa Medical Complex Gaza Palestine; ^17^ Al‐Ahli Arab Hospital Gaza Palestine; ^18^ Baylor College of Medicine Houston Texas USA; ^19^ University of Minnesota Medical School Minneapolis Minnesota USA

**Keywords:** conflict ethics, disaster ethics, Gaza, moral injury, occupational stress, psychosocial impact

## Abstract

**Background and Aims:**

Prolonged blockade, repeated attacks on health facilities, and extreme resource scarcity place Gaza's clinicians in situations that routinely violate core professional and personal values. We measured the prevalence, character, and functional consequences of moral injury among health‐care workers who have remained in Gaza since hostilities escalated in October 2023.

**Methods:**

A cross‐sectional online survey incorporated a 6‐item events subscale adapted from the Moral Injury Events Scale (MIES) and an 8‐item outcomes subscale adapted from the Moral Injury Outcome Scale (MIOS) was distributed through professional and social‐media forums from November 23, 2024, to June 1, 2025. Primary outcomes were mean MMIEOS event and outcome scores; secondary outcomes were the proportions endorsing prespecified morally injurious experiences. Descriptive statistics and Spearman correlations assessed associations with age, gender, volunteer status, and surgical role.

**Results:**

One hundred forty healthcare workers (60.7% male; mean age 24.9 ± 5.6 years; 70% unpaid volunteers) represented 36 hospitals and additional health facilities across all five governorates. Event scores were highest for witnessing immoral acts (mean 3.89 ± 1.79) and betrayal by leadership (3.36 ± 1.77). Self‐transgression items scored lower (2.55–2.84). Seventy‐six percent had witnessed colleagues' moral violations; 47% felt personally betrayed; 28% reported acting against their own values. Outcome scores demonstrated pronounced trust‐violation sequelae (mean subscale 18.08 ± 5.7 of 30) compared with shame‐related sequelae (4.38 ± 2.3 of 10). The most frequently endorsed consequences were loss of faith in humanity (mean 3.27 ± 1.50) and pervasive disgust (3.70 ± 1.33); only 14% agreed they no longer believed in a higher power. Correlations between MMIEOS scores and demographic variables were weak (|*ρ*| < 0.20, *p* > 0.05).

**Conclusion:**

Gaza's health‐care workers experience high levels of externally driven moral injury marked chiefly by violated trust and disillusionment with institutions rather than self‐directed shame. Persistent moral injury threatens workforce retention and patient‐care quality in a system already on the brink of collapse. Interventions that protect medical neutrality, ensure material support, and offer structured moral‐repair programmes are urgently needed to safeguard clinicians' psychological integrity and the sustainability of Gaza's health sector.

## 1 Introduction

Numerous iterations of the Hippocratic Medical Oath have emerged since 400 B.C., with the memorable phrase “First do no harm” (Latin: *primum non nocere*) holding sway in contemporary discourse [1]. But the practice of medical care has become riddled with an increasing burden of obstacles of various sorts, financial, legal, physical, and perhaps one of the less easily answered ones, those of an ethical nature [2, 3]. In the healthcare literature, the term “moral distress” has been noted to have risen to prominence with the seminal work of Andrew Jameton [4]. Writing in 1984, he defined moral distress along the lines of the painful psychological disequilibrium that arises when a healthcare provider is forced to act (or prevented from acting) in ways that contravene deeply held moral values [4, 5]. For instance, a nurse who believes strongly in truth‐telling may be instructed by a family member not to disclose a terminal prognosis or diagnosis to a patient, and the hospital practices may be in support of the family's request. The nurse might feel a sense of being morally “shackled,” longing to uphold patient autonomy while being told to comply with a specific cultural or institutional norm. Although individual factors might play a role, moral distress within healthcare settings is often attributed to systemic reasons and could impact any clinician regardless of profession [6, 7]. Over time, these ongoing acts against one's conscience can culminate in moral residue: a persistent sense of compromised integrity that can crescendo into moral injury [8, 9, 10].

Moral suffering is not an all‐or‐nothing phenomenon [11, 12]. Rather, it can span a continuum that may begin with moral stress, those everyday conflicts of duty and values that are eventually resolvable, and extend to moral distress, the state in which structural or personal constraints actively prevent clinicians from implementing an ethically preferable action [13, 14, 15]. Finally, at the far end of the spectrum, moral injury may erupt [16, 17]. While moral distress might manifest as tension, frustration, or heartbreak at having to follow a certain protocol one disagrees with, moral injury can represent a more corrosive and long‐lasting rupture [18]. It can involve a betrayal of what is held as profoundly sacred, fueling guilt, shame, or a sense of profound disorientation that can upend the very identity of the clinician [19, 20]. Unlike moral distress, which is often discussed in the hospital ethics literature around end‐of‐life care, resource limitations, or paternalistic policies, moral injury has been more thoroughly explored in military contexts, often focusing on combat veterans who witnessed or participated in acts that defied their moral compasses [21]. Many of these may be coerced, not chosen, a nuance stressed by certain models of moral injury [22].

The concept of moral injury draws significantly from the works of Jonathan Shay and Brett Litz [23, 24]. In their original formulation, moral injury was strongly associated with three elements: (a) the perpetration of, failure to prevent, or witness to serious moral transgressions, (b) a sense of betrayal by those in power, and (c) a pressing need to reconcile these experiences with one's moral and spiritual worldview. Military veterans, for instance, might struggle with the memory of accidentally or intentionally harming non‐combatants [24]. The moral anguish can be so debilitating that typical treatments for post‐traumatic stress disorder (PTSD) often prove insufficient because the root injury may often stem less from fear and more from shame or guilt over having violated a deeply cherished moral principle.

While moral injury was once discussed primarily in the context of warfare, interest in the phenomenon has burgeoned in civilian healthcare environments, especially in high‐stakes, resource‐deprived regions. A recent meta‐analysis reported a pooled prevalence of clinically relevant moral injury of 45% among healthcare professionals, with high exposure to potentially morally injurious events across occupations [25]. A systematic review on moral injury and mental health outcomes in nurses found significant associations between moral injury, anxiety, and depression, as well as a negative association with the quality of life [26]. Clinicians might witness or be compelled to carry out interventions that seem inhumane or inappropriate, such as turning away uninsured patients in a health system driven by profit, administering aggressive treatment that one judges to be futile, or ignoring or overriding a patient's expressed wishes due to hierarchical hospital protocols [27, 28]. Over time, these prolonged violations of the moral code can accumulate, leading to moral distress which, if unaddressed, eventually can fester into moral injury. The threshold between mere distress and deeper injury may be crossed when clinicians begin to internalize the events in global, stable, and internal ways, thereby viewing themselves as “bad people” or unworthy of redemption. This is furthered by the “social‐cultural wounds of war” that can often call for communal, not just individual, repair [22].

Moral distress can be experienced when healthcare professionals are unable to do for their patients what they feel is ethically correct, or are unaware of what is the optimal thing to do in a given scenario [29, 30, 31]. Prolonged feelings of moral distress can lead to moral injury, a profound sense of betraying one's own ethical code due to extrinsic factors and is amplified in those who bear witness to immense cruelty and human suffering [13, 16, 32]. While multiple barriers exist to optimal healthcare delivery, one at the forefront are the challenges emerging from care in a conflict or disaster affected region in the presence of limited resources [33, 34, 35, 36]. At these worst of times, when bombs are dropping, essential resources are lacking, and medical neutrality codes are brutally violated in Gaza, healthcare workers find themselves caught in situations that are fundamentally at odds with their moral principles and beliefs. It is worth noting that in this context we utilize the term medical neutrality to refer to protections under international humanitarian law requiring respect for medical personnel, facilities, and transports, and the unimpeded provision of impartial care to the wounded and sick without punishment or interference.

The impetus to explore moral injury among clinicians in Gaza is driven by both humanitarian necessity and academic responsibility [37]. The literature on moral injury, initially tethered to combat veterans, has begun to cross‐pollinate with healthcare discussions on moral distress and ethical dilemmas. This cross‐fertilization behooves a more systematic approach to understanding how extreme conditions, like mass displacement, frequent threats to personal safety and healthcare facilities, absence of basic medical resources, disruption of normal healthcare operations, an increase of theft and gang‐crime related injuries, and the forced complicity in what could be characterized as unethical care rationing, can cause or exacerbate moral injury.

Gaza's predicament creates the perfect breeding ground for moral injury within the clinical sphere [38]. Since October 2023, the Gaza Strip has been reeling under one of the most intense military operations in recent history, and the most devastating in living memory for Palestinians [39]. Tens of thousands of Palestinian civilians have been killed or injured, and nearly the entire population has been displaced multiple times, scurrying from makeshift shelters to overcrowded school buildings and back again, with no safe haven in sight [35, 40, 41, 42]. Even before this military crisis, Gaza was described as an “open‐air prison,” besieged by severe economic stagnation, political tensions, inequitable resource availability, and psychological traumas endured across entire generations [35, 43]. Restrictions on the flow of medical supplies, electricity, means of sanitation, and clean water predated the current escalation, but were severely exacerbated when hostilities reached new peaks [35]. In the midst of this chaos, healthcare workers live on the front line, not only as healers aiming to uphold medical neutrality, but as human beings trapped in an environment where moral choices are perpetually tested on a daily basis [44]. One study recently noted that nearly ~90% of Palestinian and international healthcare workers who worked or volunteered in Gaza amid the recent escalations displayed symptoms of severe depression, anxiety, and stress [45].

The scale of destruction amid the Israeli military assault on Gaza has been staggering given the continuous attacks on healthcare facilities and personnel. Major hospitals like the Al‐Shifa Medical Complex, Kamal Adwan, and dozens of other hospitals have faced bombardments, encirclement, and forced evacuations [46]. Ambulances en route to pick up casualties have been attacked, and in some reported incidents, healthcare workers themselves have been detained, injured, or killed while trying to save lives [47, 48, 49, 50]. A report from September 2024, published by Middle East Eye, mentions 1151 healthcare professionals being killed by Israeli military strikes [51]. At least 165 of those killed were physicians, 260 nurses, 300 health management and support personnel, 184 health associate professionals, 76 pharmacists, and 12 other health workers. According to multiple reports by the United Nations, the World Health Organization, and international human rights organizations, the systematic targeting of healthcare facilities in Gaza, coupled with the severe blockade preventing adequate fuel, medicine, and surgical supplies, has left the local health system on the brink of collapse [52, 53, 54]. In the face of such ethical calamity, clinicians are compelled to make agonizing decisions: who receives the scarce ventilator? Which patient do they prioritize for the limited ICU beds? Whom to prioritize or save first, should there be distinctions based on their profession, involvement in certain sectors, or the method of injury (blast wound vs. gunshot)? Is it ethically permissible to obey an evacuation order if, by leaving, they abandon the fragile patients who simply cannot be moved or a newborn in the incubator? Is it morally acceptable to not perform a limb salvage procedure that requires a lengthy duration of time, in the aim to preserve energy, surgical theater capacity, and supplies for *possibly* more important and life‐saving interventions arising from an expected mass‐casualty event?

This paper explores moral injury and its outcomes among healthcare workers in Gaza who confront daily ethical impasses of extraordinary magnitude, shaped by an unending military blockade and siege, intermittent yet devastating military assaults, and the ongoing destruction of their medical infrastructure in the most recent escalated crisis with limited provision of medical aid [55]. The pursuit is not merely academic but also pragmatic, given that moral injury is linked to an array of detrimental outcomes, including high turnover rates, mental exhaustion, erosion of empathy and suicidality, potentially decreasing the healthcare force in an already severely underserved area [56, 57]. Shedding light on these phenomena might spur targeted interventions, urgent ethical discourse and possibly policy changes, which are crucial if we are to safeguard both the frontline workers and the human lives for whom they care.

Ultimately, our aim is to address a glaring gap: the urgent need to understand the moral and psychological toll placed on those who, day after day, attempt to practice medicine amidst the gravest of human tragedies and great risk to one's own life. The data presented here seeks to highlight the moral suffering sustained by: (1) measuring the prevalence of moral injury in a sample of healthcare professionals working amidst the 2023–2025 escalations of the conflict in Gaza, (2) assessing the experiences of morally distressing or injurious events, and (3) measuring the impact on the health care professionals moral injury functional outcomes. The study also seeks to assess the association between morally injurious events and moral injury outcomes, and to explore whether event/outcome scores vary by demographic and professional factors.

## 2 Methods

We conducted a cross‐sectional online survey of healthcare workers practicing in the Gaza Strip during the period of intensified hostilities following October 2023 using convenience‐sampling. It serves as a sort of pilot study into assessing morally injurious events in the besieged enclave. This study received ethics approval from the Helsinki Committee in Gaza (PHRC/HC/1189/24).

### 2.1 Measures

There is a paucity of questionnaires assessing moral injury in medical professionals working in conflict zones, thus leading to an extensive literature review through major databases, including PubMed, Scopus, Web of Science, ScienceDirect, Embase, and APA Psychnet. We identified two validated questionnaires used mainly in the military population that could be adapted to assess moral injury among healthcare workers in the Gaza Strip during the ongoing military assault: the Moral Injury Event Scale (MIES) and the Moral Injury Outcome Scale (MIOS) [58, 59]. The MIES is a psychometrically sound measure of potentially morally injurious experiences (PMIEs) [58, 60]. It is a self‐reported questionnaire rated on Likert scale (1 = strongly disagree to 6 = strongly agree) with a higher total score indicating greater exposure to PMIEs, signifying a higher level of moral injury. It originally consisted of nine items, which were modified to six items to better suit the context of healthcare workers in an active conflict zone rather than military personnel.

The MIOS is a self‐report questionnaire developed by the Moral Injury Outcome Consortium, an international group of researchers and clinicians. The MIOS was designed to capture the severity of moral injury outcomes in response to potentially morally injurious experience [59]. It consists of 14 items, though as shortened and modified to eight items scored on a 5‐point Likert scale based on contextual relevance. The instructions provided to participants were: “Please indicate your level of agreement or disagreement with the following statements.”

Higher scores indicate more functional impairment. There are currently no scoring categories or cut‐offs. The currently used MIOS was adapted from it for ease of administration and relevance. The MIOS consists of two subscales. Subscale 1, which measures shame‐related outcomes, comprises items 1 and 5, with a possible final score range of 2–10 upon adding individual responses. Subscale 2, assessing trust violation‐related outcomes, includes items 2, 3, 4, 6, 7, and 8, with a possible score range of 6–30 when summing up individual scores of the six questions.

### 2.2 Participants

The MMIEOS was distributed as a Google Form among healthcare workers in the Gaza Strip using several medical institutional, professional and healthcare‐related social media groups. Due to the absence of centralized or official platforms uniting all healthcare workers in Gaza by specialty or affiliation, a precise response rate could not be calculated. The primary eligibility criteria were: age ≥ 18 years, current or recent clinical role since October 2023 in Gaza, and consent to participate. Exclusion criteria were: (1) no consent, (2) not meeting the Gaza clinical‐role criterion, or (3) invalid/duplicate records when identifiable.

Demographic and professional variables collected included age (years), gender (male/female), primary governorate of practice during the prior year, role(s) since October 2023, employment status (volunteer/fixed/contract/board training), self‐reported adequacy of financial compensation (yes/no/prefer not to say), years of experience, performance of surgically invasive procedures (yes/no), and facility/facilities served.

Data collection occurred in two waves: the first targeted professional groups, and the second leveraged social media groups. The form was open from November 23, 2024, to June 1, 2025, during which 140 responses were collected. All completed forms were included in the final analysis. Demographic data were also collected.

### 2.3 Data Analysis

Descriptive statistics, including means, standard deviations (SDs), and ranges, were calculated for all collected data. To explore potential relationships between demographic variables and scale items, a correlation analysis was conducted. As the form was required to be fully completed to be processed and recorded, there was no missing data to account for.

Data were analyzed using R version 4.4.2. Continuous variables are presented as mean ± standard deviation (SD) and range; categorical variables are presented as frequencies and percentages. Because item‐level responses to the survey scales are ordinal, associations between demographic/professional factors and event/outcome scores were evaluated using Spearman rank correlations (ρ). All statistical tests were two‐sided, and statistical significance was defined a priori as *p* < 0.05. Given the exploratory pilot design, analyses were interpreted as hypothesis‐generating and no multiplicity correction was applied.

For analysis, the moral injury events score was calculated as the mean of the six adapted event items (possible range: 1–6). The moral injury outcomes score was summarized both by item‐level means and by subscale totals (shame‐related: items 1 and 5, range 2–10; trust‐violation‐related: items 2, 3, 4, 6, 7, and 8, range 6–30), with higher scores indicating greater symptom burden.

## 3 Results

The survey was filled out by 140 healthcare professionals. Table 1 describes the demographics of the healthcare professionals. Seventy percent of the respondents were volunteers, with the mean age of 24.86 years, an average experience of 1.91 years, and males comprising 60.7% of the sample.

**TABLE 1:** Demographic and professional characteristics of respondents.

Characteristics	Value (*n* = 140)
Age (years)	Mean = 24.86 + /− 5.57
	Range = 18–54
Gender	Male: 85 (60.7%)
	Female: 55 (39.3%)
Location*	North Gaza: 12 (8.6%)
	Gaza: 26 (18.6%)
	Deir Al‐Balah: 37 (26.4%)
	Khan Yunis: 60 (42.9%)
	Rafah: 5 (3.6%)
Performed surgically invasive procedures	42 (30%)
Employment	Volunteer: 98 (70%)
	Fixed: 18 (12.9%)
	Contract: 15 (10.7%)
	Board training: 9 (6.4%)
Adequately financially compensated	Yes: 7 (5%)
	No: 122 (87.1%)
	Prefer not to say: 11 (7.9%)
Years of experience	Mean = 1.91 years
	Range = 0–35 years
Role**	Medical student (80)
	Nursing student (2)
	Intern (physician) (19)
	General practitioner (physician) (38)
	Master's degree (physician) (2)
	Resident (physician) (13)
	Consultant (physician) (2)
	Nurse (23)
	Anesthesia technician (1)
	Physiotherapist (1)
	Medical laboratory specialist (1)
Hospitals***	Al‐Adwa Hospital‐North (4)
	Al‐Karama Specialized Hospital (1)
	Beit Hanoun Hospital (3)
	Indonesian Hospital (9)
	Kamal Adwan Hospital (7)
	Al‐Ahli Arab Hospital (28)
	Al‐Helou International Hospital (3)
	Al‐Ranteesi Al‐Naser Paediatric Hospital (2)
	Al‐Rantisi Specialized Hospital (1)
	Al‐Shifa Medical Complex (36)
	Al‐Wafaa Rehabilitation Hospital (1)
	Haifa Charity Hospital (1)
	Patient Friends Association Hospital (1)
	Psychiatric Hospital (1)
	Public Aid‐Cardiovascular Hospital (1)
	Al‐Aqsa Martyrs Hospital (35)
	Al‐Awda Hospital‐Nuseriat (8)
	Yaffa Hospital (1)
	Al‐Amal Hospital (1)
	European Gaza Hospital (26)
	Nasser Hospital (54)
	Al‐Helal Al‐Emirati Hospital (8)
	Al‐Najjar Hospital (10)
	Kuwait Hospital (7)
	Other clinics (19)

* Participants were asked to note which governorate they spent most of the last year in.

** The question asked respondents to report all the roles they occupied since October 7, 2023, accounting for role transition, so the total *n* > 140.

*** The question asked respondents to report all the facilities they served at amid the recent escalations, so the total *n* > 140.

Upon review of the descriptive results of MIES presented, the items assessing transgressions of moral code by others (Q1 and Q2) and betrayal of trust (Q6) exhibited higher mean scores (> 4 on a 6‐point scale) compared to items evaluating transgressions of moral code by self (Q3, Q4, and Q5), which demonstrated lower mean scores (< 3) (Table 2).

**TABLE 2:** Descriptive statistics for the moral‐injury events items (1 = strongly disagree to 6 = strongly agree).

	Moral injury statement	Mean	SD	Range
1	I saw things that were morally wrong	3.89	1.79	1–6
2	I am troubled by having witnessed others' immoral acts	3.79	1.75	1–6
3	I acted in ways that violated my own moral codes/values	2.55	1.69	1–6
4	I violated my own morals by failing to do something I felt I should have done	2.75	1.59	1–6
5	I am troubled because I violated my morals by failing to do something I felt I should have done	2.84	1.63	1–6
6	I feel betrayed by leaders who I once trusted	3.36	1.77	1–6

The results of the MIOS indicate that the mean scores for trust violation‐related outcomes (18.08 out of 30) were notably higher than those for shame‐related outcomes (4.38 out of 10), suggesting a greater emphasis on trust violations in the context of the measured constructs (Table 3).

**TABLE 3:** Moral injury outcome items (1 = strongly disagree to 5 = strongly agree).

	Moral injury outcome	Mean	SD	Range
1	I blame myself	2.37	1.27	1–5
2	I have lost faith in humanity	3.27	1.50	1–5
3	I have trouble seeing goodness in others	2.78	1.38	1–5
4	I am disgusted by what happened	3.70	1.33	1–5
5	I feel like I don't deserve a good life	2.01	1.40	1–5
6	I no longer believe there is a higher power	2.39	1.47	1–5
7	I lost trust in others	3.09	1.34	1–5
8	I am angry all the time	2.85	1.31	1–5

**FIGURE 1:**
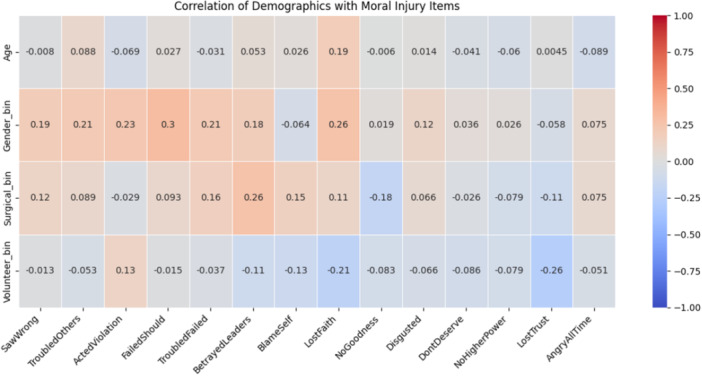
Moral injury statements correlations.

In Figure 1, the bottom three rows are binary variables where “Gender_bin” is defined as 1 if the form‐filler is a male and 0 if they're a female; “Surgical_bin” is 1 if the respondent is a surgeon or performed surgically invasive procedures (0 if not); and similarly, “Volunteer_bin” is defined as 1 if the participant is a volunteer and 0 otherwise. None of the correlations appears at the outset to be particularly strong.

An additional three self‐report questions on stressful experiences were asked, summarized in Table 4.

**TABLE 4:** Experiences you had after a stressful event that is currently most distressing.

Question	*N*	%
Did something (or failed to do something) that went against your moral code or values	39	27.9
You saw someone (or people) do something or fail to do something that went against your moral code or values	106	75.7
You were directly affected by someone doing something or failing to do something that went against your moral code or values (e.g., being betrayed by someone you trusted)	66	47.1

## 4 Discussion

### 4.1 Implications of the Israeli Military Assault on Gaza

The infrastructure in the besieged enclave is shattered by repeated assaults [61]. Hospitals continue to run out of fuel amid delays or pauses in aid deliveries, forcing staff to choose which essential services remain operational [62]. The water supply is contaminated as reservoirs and purifiers have been destroyed [63]. Israeli soldiers sometimes roam through wards, carrying out raids or arrests [64]. Airstrikes pulverize entire neighborhoods, instantly flooding the nearest emergency department with hundreds of casualties, far exceeding the capacity of even the best‐staffed hospital [65]. Meanwhile, foreign aid gets stuck at sealed borders or looted by gangs facilitated by Israel, leaving local staff to operate with painfully inadequate supplies of essential drugs and devices [66]. This scenario imposes a profound moral burden on providers who, day in and day out, are forced to watch helplessly as patients die from treatable injuries or diseases simply because the basic prerequisites for life‐saving care are denied [67]. Over time, the repeated witnessing of such preventable tragedies can corrode one's sense of professional identity and moral wholeness. Healthcare personnel speak of feeling like powerless bystanders, witnessing a system they are part of failing to do what it was meant to, which is to save lives, on a routine basis. Some feel as if they are part of a normalized cycle of death, in direct opposition to what they were trained to do.

The results of our pilot study provide a useful lens to peer into the impact of the crisis on Palestinian healthcare workers in Gaza. Only 5% of survey participants expressed feeling adequately financially compensated, which represents a testimony to the disruptions of cash‐flow, economic meltdown, hyperinflation and stagflation, and the utter collapse of the market, jobs, and any semblance of a working‐life and livable conditions [68, 69, 70]. A striking fact worth highlighting is that the majority of the respondents were volunteers. Even though they were willing to provide services without any compensation, relying on their labor is not sustainable long‐term given there are no signs of resolution and repair despite the current atrocities extending over a year. Healthcare professionals working in high‐intensity or crisis contexts invariably face a convergence of moral distress, resource limitations, and organizational shortfalls that heighten the risk of burnout, decreased job satisfaction, and potential moral injury [71]. Various studies show that interventions aimed at improving working conditions (e.g., optimal staffing levels, fair compensation, and robust safety culture) are pivotal in reducing burnout, fostering retention, and enhancing patient care outcomes, all of which are difficult if not impossible in the context of Gaza [72, 73]. From a policy perspective, compensating caregivers equitably not only alleviates financial stressors but also signals institutional appreciation of their expertise and dedication, a challenging ordeal for the Ministry of Health in Gaza as finances are under extreme strain while numerous volunteers feel inadequately compensated [74]. Indeed, this mirrors findings in broader disaster or emergency‐affected populations, where the psychosocial impacts of under‐compensation and prolonged occupational stress include heightened intent for job‐turnover, reduced engagement, and compromised well‐being [75, 76]. Improved compensation strategies ranging from wage increases, expansions of benefits, and transparent promotion opportunities, have consistently emerged as potent buffers against chronic stress, emotional exhaustion, and overall burnout, and may be avenues to explore in a setting where the crisis de‐escalates and economic flow increases [74]. However, compensation alone is insufficient: studies highlight that employees who experience supportive leadership, harmonious team relationships, and meaningful opportunities for career growth display significantly lower rates of depersonalization and higher rates of career longevity, underscoring that conducive work environments and a sense of vocational commitment serve as equally critical drivers of workforce stability [73]. This is something that must be considered as strong healthcare retention will be needed for the next several decades in Gaza given the destruction of medical infrastructure, teaching hospitals, universities, and the killing and abduction of specialists [77].

Evidence also further indicates that willingness to remain in the same career path mediates the relationship between compensation and burnout, effectively magnifying or diminishing the impact that enhanced pay structures can have on day‐to‐day resilience [74]. In other words, a fairly compensated caregiver who envisions a long‐term trajectory in the profession is less likely to succumb to the emotional fatigue and cynicism that often trigger turnover [76]. Although improved pay is undeniably important, organizational measures such as structured mentorship, robust mental health support, and participatory governance also do play a complementary role in attempting to protect caregivers against the corrosive effects of moral distress and prolonged occupational pressures [71]. Taken together, these findings from our study show a dual imperative for policymakers and healthcare organizations that will operate in Gaza when the crisis de‐escalates, and to some extent even now if possible: first, to raise compensation and benefits in a manner commensurate with professional competencies, and second, to implement systemic improvements (e.g., supportive leadership and reasonable workloads) that fully leverage the protective effects of fair compensation and foster a psychologically healthy workforce [72].

With the claim, “I no longer believe there is a higher power,” 60/140 (~ 43%) respondents reported that they strongly disagreed with the statement, that number rises to 80/140 (~ 57%) when accounting for all disagreements. In contrast, 20/140 (~ 14%) of respondents strongly agreed with the claim, while an additional 15/140 (~ 11%) agreed with it. This was the second‐most uniform result. The minority of respondents who agreed or strongly agreed with “I no longer believe there is a higher power” points to a subset for whom the horror of witnessing mass suffering, such as the preventable deaths and bombardments described in Gaza, seems to have eroded their faith in a transcendent force [74, 78]. In contexts of extreme helplessness, moral outrage, and the crushing devastation of war that is genocidal and prosecuted as a genocide, religious disillusionment can set in when conventional tenets of meaning‐making fail to reconcile the magnitude of the unfolding tragedy [39, 54, 79, 80]. Familial dynamics, many of which have been disrupted in Gaza due to loss of relatives, can partly shape the trajectory of religiosity and mental well‐being, but robust longitudinal findings suggest a persistent, if attenuated, relationship even when family background is accounted for [81]. This disillusionment is further corroborated by empirical work showing how negative, prolonged, and seemingly inexplicable trauma may give rise to “spiritual struggles,” a process chaplain‐led, bio‐psycho‐social‐spiritual models regard as central to healing, and the idea that the God or divine entity one chooses to believe in is indifferent or has abandoned them [82, 83, 84]. It is worth noting that the fraction of respondents who lost faith here, although less numerous compared to those who remain steadfast or even potentially turn more strongly to faith, reveals that acute stressors, violent deaths, and a protracted sense of betrayal can trigger a complete renunciation of previously held beliefs [85, 86]. Religious affiliation data were not collected in this study. Relatedly, an analysis of German panel data shows how adverse events, particularly the death of a spouse, often leads to higher religious activity [87]. Additionally, given that over two‐thirds of the sample were volunteers, and no one reported being adequately compensated, there may be an intersection between economic insecurity and potentially compounding moral injury. By parallel, research during COVID‐19 suggests that greater reliance on God helps mitigate psychological distress when financial strain is high [79]. No data was collected on the number of deceased relatives, loved ones, or colleagues for healthcare workers in this study.

Nevertheless, this phenomenon does not negate the broader observation that the majority of respondents either strongly rejected or overall disagreed with the statement “I no longer believe there is a higher power.” In direct defiance of the extraordinary terror experienced, many Palestinians report feeling an intensification of their commitment to God amid the calamities befalling them [88, 89]. Others have reported acquiring a newfound interest in Islam, or even converting, as a consequence of the strong belief in God exhibited by Palestinians following airstrikes, death, and loss of loved ones and family members [90, 91, 92, 93, 94, 95]. This many times has manifested in oft‐circulated videos of survivors stating “*al‐ḥamdu li‐llāh*” (all praise and gratitude are for Allah), “*al‐ḥamdu li‐llāh ʿalā kulli ḥāl*” (all praise and gratitude are for Allah in every circumstance), or “*ḥasbunā llāhu wa ni'ma l‐wakīl*” (Allah alone is sufficient for us, and He is the best Disposer of affairs) [96]. Multiple studies, including those conducted in Palestine, confirm that religion frequently serves as a pivotal psychosocial resource, particularly in high‐distress or conflict settings, by offering a sense of moral coherence and emotional resilience through prayer, communal worship, or reliance on God [80, 97, 98, 99, 100]. Nevertheless, an individual's experience of relentless or egregious injustices, such as the ongoing trauma emerging from genocide in Gaza and the loss of loves ones and society, can invert the usual coping mechanism of faith, heightening moral injury or inciting a sense of cosmic betrayal if religious frameworks are unable to alleviate or justify the suffering observed in a given individual's own conceptualization [101]. Loss of faith can also mirror the crisis‐of‐meaning pattern described in multi‐faith injury care studies [102]. Definitional debates on “spiritual injury” vs. moral injury reinforce this point [103]. In effect, while religiosity often flourishes under adversity, a contingent portion of people confront “fatality forming,” in which they view the catastrophic events as evidence that no benevolent higher power exists [78, 104]. For the statement, “I feel like I don't deserve a good life,” 87/140 (~ 59%) of respondents asserted that they strongly disagreed with the proposition. This was the most decisive response result for any single question in the entire study. It stands as a testimony to the perseverance of the healthcare workers in Gaza against all odds, and the general resilience that has been displayed time and again by a population enduring decades of occupation, siege, and apartheid. Generally, no significant differences were found between any of the response statements when stratified for age, gender, volunteer status, or having performed surgically invasive procedures. This lack of variance in responses across demographic and professional variables likely reflects the overwhelming and consistent psychological impact of intense and collective exposure to moral injury and terror, where individual characteristics, such as age, gender, or professional role, are overshadowed by the shared extremity of the experience.

### 4.2 Moral Distress, Injury, and Fatigue in Gaza

Some might regard “moral distress” and “moral injury” as interchangeable, but there are nuanced distinctions that do exist. Moral distress often emerges from ethically challenging but routine situations, such as providing non‐beneficial care to a terminal patient because of family insistence or feeling overwhelmed by staffing shortages that compromise the delivery of safe and compassionate care. These experiences, while painful and ethically disorienting, typically remain at the level of acute distress or frustration and can be mitigated through supportive institutional policies, debriefing sessions, strong mentoring from senior colleagues, or other moral resilience practices [19]. Moral injury, by contrast, sets in when these constraints and conflicts are persistent, severe, and in direct opposition to one's foundational moral beliefs. This might manifest as a deep, gnawing guilt for having to triage wounded civilians to “expectant” status, essentially designating them as beyond help even though with advanced resources, they might have survived. Or it might emerge from a sense of having “betrayed one's oath” in not speaking out against systematic violations of medical neutrality more forcefully, be that not taking interviews with journalists or reporting out about it for fear of retaliation or detention by Israeli military forces.

When moral injury does occur, it is associated with serious psychosocial consequences. Research in the military domain has documented associations between moral injury and increased suicidal ideation, heightened risk of self‐harm, persistent shame, difficulties maintaining personal relationships, and a diminished sense of self‐worth [105]. It is worth highlighting however that disagreement with statements such as “I feel like I don't deserve a good life” is reflective of the potentially distinct ways in which healthcare providers manifest moral injury, given hope and self‐appreciation may still exist. This may be rooted in the profession's responsibility to save lives, in contrast to a military domain where one participates in, or treats, active combatants. Among healthcare workers, these manifestations align with well‐known themes of burnout, emotional exhaustion, secondary trauma, compassion fatigue, and in extreme cases, an exodus from the profession altogether and suicidal tendency [106, 107]. Preliminary studies in conflict zones demonstrate that moral injury compounds the psychological burden of frontline clinicians, heightening rates of depression, anxiety, and post‐traumatic symptoms beyond what would be expected from the direct stress of conflict [108]. Such individuals may also become cynical, harboring a pervasive mistrust in institutions and even colleagues, undermining the collaborative fabric required in any effective healthcare system. In acute conflict settings like Gaza, where the boundaries between professional duty and personal survival blur, moral injury has the potential to reverberate through entire hospital wards, eroding team spirit and further diminishing the quality of care provided to patients.

One conceptual framework that might prove invaluable draws on existing models from the military domain, such as the intersection of “acts of commission or omission,” “a sense of betrayal,” and “difficulty forgiving oneself or others,” all culminating in profound psychological or spiritual anguish. But for the healthcare context, especially in a long‐blockaded conflict zone like Gaza, one must also layer in the complexities of professional codes of ethics, local cultural norms, and its intersection with international humanitarian law. Institutional betrayal in humanitarian spaces likewise deepens moral injury, echoing findings from decolonizing‐aid analyses [109]. Ulrich & Grady's international survey of moral distress offers insights into other global patterns for comparable investigations [110]. We suspect that moral injury among healthcare workers in Gaza likely exhibits unique features, including an ingrained sense of repeated betrayal by global governance bodies or the international community. Many local clinicians feel that if the atrocities were occurring elsewhere, urgent interventions might have already been implemented. As such, the moral injury they experience may be a swirl of guilt, anger, and disillusionment not only at the local level (e.g., battered facilities) but at a larger geo‐political scale.

### 4.3 Limitations

While the study offers useful insights into what is occurring in the moral‐psychosocial realm for healthcare workers in Gaza, it is limited by its small sample size. Evidently, the historical context of armed conflict and victims of violence may influence who is completing the survey and the responses themselves, with some studies indicating that localized bombings can reduce response rates, as well as longer questionnaires [111]. Internet and power outages, frequent displacement events coupled with airstrikes, mass‐casualty events at hospitals, resource scarcity, lack of usual provisions, destruction of medical facilities, breakdown of medical infrastructure, and most of all, the loss of lives of numerous healthcare professionals are some of the factors which led to low recruitment. The study was also not disseminated through any one central platform, instead relying on a plethora of ad hoc groups to reach a broader audience. It was also not possible to properly contextualize how representative this sample was as a proportion of all the healthcare workers in Gaza, despite recording the hospitals one served at, as the total span of healthcare facilities and workers at a given time is constantly in flux due to repeated displacement and strikes against them. Furthermore, the questionnaires used were modified from established surveys which can compromise the validity and potential applicability for the Gaza‐specific context, but due to the lack of any pre‐existing relevant survey one had to be tailored to this unique cohort of medical professionals working amid such dire conditions. Some of the questions, such as “I no longer believe there is a higher power” also risked being interpreted differently: as “higher power” is not the commonly‐used term to refer to God or a divine entity in Palestine, English is not the first‐language of most Palestinians, and the Western milieu's cultural context‐specific usage of terms may not be understood the same way by a different population. It is quite plausible that the term was interpreted by some to mean international agencies/intervention, governmental authorities, or the moral “goodness” of the global community claiming to champion human rights, among other possibilities.

### 4.4 Considerations for Professional Practice and Service Quality

The main pattern in our data is one of externally driven moral injury, characterized more by witnessing transgressions and institutional betrayal than necessarily by self‐directed shame. In practice, this matters because trust rupture, chronic disgust, and persistent anger may degrade team communication, weaken interprofessional trust, and reduce continuity of care. In fragile health systems, these pathways can translate into poorer quality outcomes through increased absenteeism, diminished workforce retention, and higher risk of errors during high‐acuity decision‐making. There is also the need to pay attention to the possibility of high levels of anxiety, depression, and stress among healthcare workers [45, 112].

Practical interventions could therefore combine psychosocial and organizational components. First, services may benefit from implementing structured post‐shift ethical debriefs and peer‐support pathways after mass‐casualty exposure (when possible). Leadership could also prioritize staffing and scheduling protections (including rest and leave access when feasible), as workplace conditions are often modifiable drivers of psychological strain and burnout. If there is enough capacity, services could provide confidential referral pathways (e.g., for mental health and spiritual care) for staff with persistent symptoms. Many of these are difficult, if not impossible, to implement during situations of active armed conflict, but may still be considerations for periods of reduced intensity in violence.

## 5 Conclusion

Healthcare workers in Gaza face a unique convergence of systematic violence against their institutions, family members, and colleagues. The findings from this study confirm both the pervasiveness and intensity of moral injury, painting a sobering portrait of how repeated ethical compromise and moral transgression, combined with a shortage of available mechanisms to cope or heal, can break the will and fracture the well‐being of some healthcare workers. The ramifications go beyond personal suffering: moral injury undermines team cohesion, patient care, and the general functioning of a healthcare infrastructure under siege. Yet amidst these grim findings there is an opportunity for hope. Identifying moral injury, recognizing its impact on healthcare professionals and understanding its prospective ramifications can spur both immediate and long‐term dialog and interventions for the rebuilding of Gaza. On the ground, small acts of moral repair, empathy, and communal support from international aid workers and medical staff can help. On a larger scale, forceful advocacy for the upholding of medical neutrality and the restoration of vital resources could be an initial small step to alleviate the conditions that breed moral injury. We must acknowledge however that moral repair in Gaza requires addressing the very root cause: genocide, apartheid, and siege that generates these impossible ethical dilemmas. Only then can we begin to treat the deep wounds inflicted not only on the human bodies but on the consciences of those who strive to heal them.

## Author Contributions

**Bilal Irfan:** conceptualization, writing – original draft, data curation, formal analysis, investigation, methodology, project administration, supervision, visualization, writing – review and editing. **Abdulwhhab Abu Alamrain:** conceptualization, writing – review and editing, data curation. **Khalil Alinaby:** data curation, writing – review and editing. **Hasan Hussein:** formal analysis, writing – review and editing. **Abdallah Abu Shammala:** conceptualization, data curation, writing – review and editing. **Mohammed Halimy:** conceptualization, data curation, writing – review and editing. **Majdi Al‐Khaldi:** conceptualization, data curation, writing – review and editing. **Alaa Jamal Kassab:** conceptualization, writing – review and editing, data curation. **Fahmy Almedana:** conceptualization, writing – review and editing, data curation. **Hitham Ibrahim Toman:** conceptualization, writing – review and editing, data curation. **Basel Tarab:** writing – review and editing. **Roberto Sirvent:** writing – review and editing. **Abd Al‐Karim Sammour:** data curation, writing – review and editing. **Hanaa Mousa:** writing – review and editing, data curation. **Belal Aldabbour:** data curation, writing – review and editing. **Izzeddin Lulu:** writing – review and editing, data curation. **Ali Ghaleez:** writing – review and editing, data curation. **Amna Abusamhadana:** data curation, writing ‐ review and editing. **Abdullah Ghali:** writing – review and editing. **Muna Irfan:** writing – review and editing, conceptualization, investigation, methodology, project administration, supervision, writing – original draft.

## Funding

The authors have nothing to report.

## Disclosure

The lead author Bilal Irfan affirms that this manuscript is an honest, accurate, and transparent account of the study being reported; that no important aspects of the study have been omitted; and that any discrepancies from the study as planned (and, if relevant, registered) have been explained.

## Conflicts of Interest

The authors declare no conflicts of interest.

## Data Availability

The data that support the findings of this study are available from the corresponding author upon reasonable request.
